# Concurrent Resolution of Chronic Diarrhea Likely Due to Crohn’s Disease and Infection with *Mycobacterium avium paratuberculosis*

**DOI:** 10.3389/fmed.2016.00049

**Published:** 2016-10-27

**Authors:** Shoor V. Singh, J. Todd Kuenstner, William C. Davis, Prabhat Agarwal, Naveen Kumar, Devendra Singh, Saurabh Gupta, Kundan K. Chaubey, Ashok Kumar, Jyoti Misri, Sujatha Jayaraman, Jagdip S. Sohal, Kuldeep Dhama

**Affiliations:** ^1^Microbiology Laboratory, Animal Health Division, Central Institute for Research on Goats, Mathura, India; ^2^Temple University Hospital, Philadelphia, PA, USA; ^3^Microbiology and Pathology, Washington State University, Pullman, WA, USA; ^4^Department of Medicine, S.N. Medical College, Agra, India; ^5^State Animal Husbandry Department, Kumher, India; ^6^Division of Animal Science, Krishi Bhavan (ICAR), New Delhi, India; ^7^Amity Institute of Microbial Technology, Amity University Jaipur, Jaipur, India; ^8^Department of Pathology, Indian Veterinary Research Institute, Bareilly, India

**Keywords:** *Mycobacterium avium paratuberculosis*, Crohn’s disease, antibiotic therapy, stool culture and microscopy for *MAP*, ELISA, IS*900* PCR and IS*1311* PCR_RE

## Abstract

Examination of samples of stool from a 61-year-old male patient, presenting with the clinical symptoms of Crohn’s disease (CD), revealed massive shedding of acid fast bacilli with the morphology of *Mycobacterium avium paratuberculosis* (*MAP*), the causative agent of Johne’s disease in cattle. *MAP* was cultured from the stool. Biotyping of the bacterium isolated from cultures of stool demonstrated, it was the Indian Bison biotype of *MAP*, the dominant biotype infecting livestock and humans in India. Based on this finding and because the patient was unresponsive to standard therapy used in India to treat patients with gastrointestinal inflammatory disorders, the patient was placed on a regimen of multi-antibiotic therapy, currently used to treat tuberculosis and CD. After 1 year of treatment, the patient’s health was restored, concurrent with cessation of shedding of *MAP* in his stool. This patient is the first case shown to shed *MAP* from the stool who was cured of infection with antibiotics and who was concurrently cured of clinical signs of CD.

## Introduction

A major question that has remained unanswered over the past years is whether *Mycobacterium avium paratuberculosis* (*MAP*), the causative agent of Johne’s disease in cattle, is zoonotic and also the causative agent of Crohn’s disease (CD). The lack of resolution of this question is partly attributable to the difficulty of demonstrating the presence of *MAP* in all patients with the clinical symptoms of CD and the finding of *MAP* in patients with other gastrointestinal disorders as well as other diseases and also in humans with no signs of disease. The case report presented here, and results from an extensive survey we recently conducted in India, have provided an explanation for why it has been difficult to show *MAP* is the causative agent of CD in, at least, a subset of patients with CD. The survey of 42,400 subjects revealed humans like other species are equally susceptible to infection with *MAP* regardless of health status (1). We detected the presence of *MAP* in subjects with no clinical signs of disease and subjects with disorders involving the skin, liver, abdomen, gastrointestinal tract, thyroid, blood (anemia), pancreas (diabetes), autoimmune inflammatory disorders, and infectious diseases, e.g., tuberculosis, typhoid, and malaria. The survey also revealed very few of subjects infected with *MAP* had detectable cocco-bacilli in their stool, as detected by microscopy, including subjects with overt clinical signs of CD, similar to an earlier study (2). The subjects identified in these studies were poor, and there was no opportunity to arrange for treatment, leaving open the question of whether antibiotic therapy could be used to cure infection with *MAP* and whether a cure would also affect a cure of CD. The subject of this case report has provided an opportunity to answer both questions.

## Case Report

**Figure 1:**
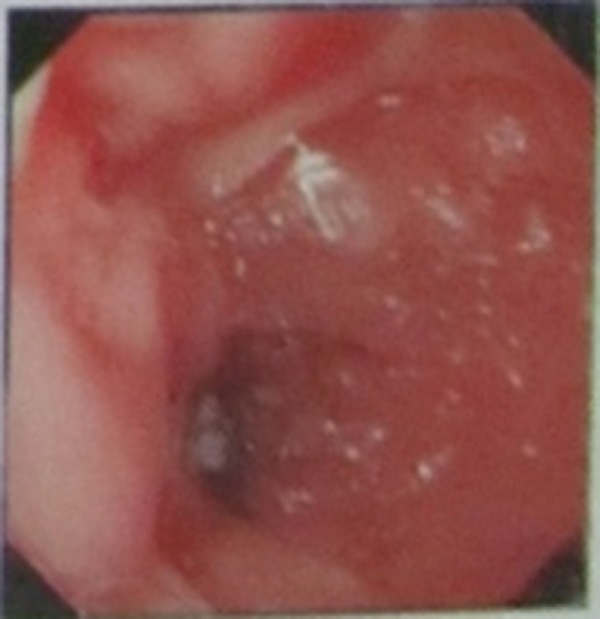
**Representative picture showing results from three independent endoscopic evaluations of the intestine of the patient obtained at different times before he was started on antibiotic therapy**. There was no opportunity for further evaluations during treatment.

A 61-year-old male, a resident of a rural village of Rajasthan state in India, had a history of frequent bowel movements with passage of loose stool or mucus every half hour. He had lost weight and was emotionally distressed by the lack of the medical community’s ability to obtain a diagnosis and treatment for his illness. The patient was referred to us at the Central Institute for Goat Research (CIRG) approximately 3 years ago by his relative, a veterinary officer in the State Animal Husbandry Department of Rajasthan state. The officer was familiar with the screening of animals for *MAP* infection in goats, sheep, cattle, and buffaloes and knew of the reported association of *MAP* with CD. On the initial visit, the patient suffered from severe depression in addition to presenting with the clinical signs and symptoms of diarrhea, weakness, weight loss, fatigue, and anorexia, symptoms that closely resemble the clinical symptoms of Johne’s disease in cattle, except for anorexia [reviewed in Ref. (3)]. At the clinical stage of Johne’s disease, there is extensive infiltration of the mucosa with *MAP* infected macrophages and lymphocytes, associated with a loss in the integrity of the absorptive epithelium, resulting weight loss, and constant diarrhea. Bacteria are readily identified in feces at this late stage of disease [reviewed in Ref. (3)]. The patient was desperate and turned to the CIRG as a last resort. The patient had consulted with gastroenterologists over the previous years and sought care in hospitals in Bharatpur and New Delhi (Aakash Medical Center, Sir Ganga Ram Hospital and All India Institute of Medical Sciences) for the treatment of his ailment. Several endoscopic evaluations conducted during the visits to hospitals demonstrated the presence of inflammation in the intestine (Figure 1); however, the gastroenterologists did not include CD as one of the possible reasons for inflammation. Biopsies were not taken for evaluation. The patient was treated with the standard therapy for Irritable bowel diarrhea (IBD), steroids, and azathioprine. Treatment provided no improvement.

**Figure 2:**
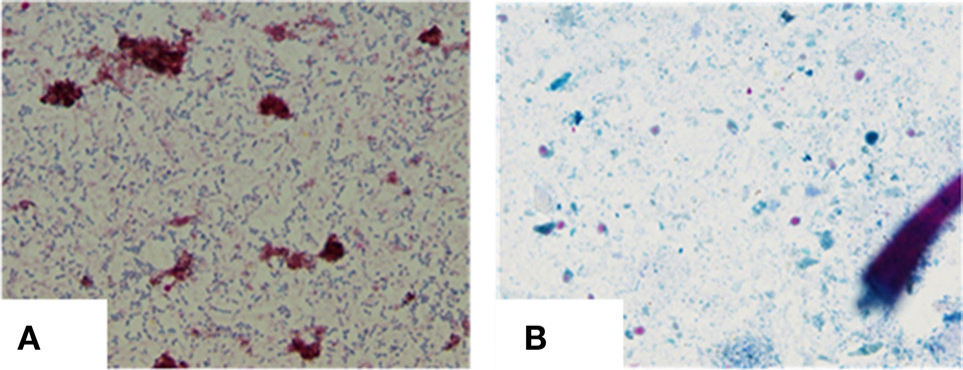
**(A)** Heavy shedding of typical *MAP* bacilli as seen in ZN staining: 4+ (positive). **(B)** Negative for *MAP* bacilli at the end of 12 months of treatment.

Serum, blood, and stool were collected to check for the presence of *MAP* infection, using the four diagnostic tests [microscopy, culture, IS*900* PCR (stool and blood), and ELISA] used in the survey (1, 4). The general procedure for isolating *MAP* for culture and examination by microscopy in our laboratory proved to be exceptionally important with this case. The procedure for screening for the presence of *MAP* in fecal samples (2) is included in this report so that this procedure may be used by other laboratories. Approximately 2 g of stool were placed in 12 ml of sterilized water and finely minced using a pestle and mortar under sterilized conditions. The mixture was then centrifuged at 4,500 rpm at room temperature for 45 min. The supernatant was discarded, leaving a pellet, comprised of a dense layer of solid material at the bottom and a thin layer of semisolid less dense material at the top. Two sterile swabs were used to collect material from the semisolid upper layer, one to make a slide and the second to prepare a culture. The latter swab was placed in a tube containing 40 ml of a 0.9% hexadecyl pyridinium chloride in water to decontaminate fecal material. The mixture was allowed to stand for 18 h at room temperature. The supernatant was decanted and 0.2 ml of the residual sediment inoculated onto slants of Herrold’s egg yolk medium (three containing mycobactin J and one without mycobactin J). Microscopic examination of the stool specimen revealed the patient was shedding massive numbers of acid fast short rods (cocco-bacilli) (+3 to +4), with morphology indistinguishable from *MAP* (Figure 2A). Following 5 months of culture of stool, small colonies of bacteria were detected in agar slants containing mycobactin J. Biotyping of the colonies with the IS*1311* PCR_RE and IS*1311* L2 PCR_REA (5, 6) demonstrated the bacterium was the “Indian Bison type,” of *MAP*, the prevalent form of *MAP* in India (4). The patient’s serum was positive for *MAP* by ELISA.

## Treatment

After our report to the patient, the patient went to New Delhi to the All India Institute of Medical Sciences, which is one of the best institutions in Medicine in India. He met with the Head of the Division of Gastroenterology for treatment. The gastroenterologist believed the patient had IBD and treated him accordingly with the standard treatment for IBD. Following 15 days of treatment, there was no improvement in the patient’s failing condition and he returned to the CIRG very depressed. He was losing weight with the frequency of bowel movements every half hour. He requested the CIRG provide him with an injection of institute’s vaccine that was shown to have a therapeutic effect. He was informed that this was not possible. As an alternative, we at CIRG arranged for consultation with a local physician and coauthor, Dr. Prabhat Agarwal, an Assistant Professor at the S.N. Medical College, Agra, with a small clinic in Farah where CIRG is located. Dr. Prabhat Agarwal took interest in the case and was willing to consider the patient’s illness could be associated with an extensive infection with *MAP*. Dr. T. J. Borody, who had reported success in treating patients with CD with antibiotics was contacted for advice and recommendation on how to treat the patient (7). Dr. Borody provided copies of recent publications and suggested what antibiotics should be considered for treatment. Based on his suggestions, a regimen of therapy was designed and implemented. The patient was informed that his clinical symptoms were most likely caused by a *MAP* infection and that a regimen of antibiotic therapy might provide a cure. After obtaining his written consent, he was placed on anti-*MAP* therapy under the supervision of Dr. Prabhat Agarwal. The antibiotic therapy included clarithromycin, rifampin, ethambutol, levofloxacin, isoniazid, and rifaximin along with mesalazine according to the schedule in Table 1.

**Table 1:** **Summary of treatment regimen up to 12 months**.

Drug categories	Type of medicine	Treatment duration
Antibiotics	Levofloxacin (750 mg)	1–11 weeks
	Clarithromycin (250 mg)	9–38 weeks
	Isoniazid (300 mg)	Up to 13 weeks
	Rifampicin (450 mg)	Up to 13 weeks
	Ethambutol (800 mg)	Up to 13 weeks
	Rifaximin (550 mg)	7–18 weeks
	Rifabutin (150 mg)	11–42 weeks
Anti-inflammatory	Mesalazine or 5-aminosalicylic acid	1–38 weeks (1 g) and up to 48 weeks (2 g)

## Results

**Figure 3:**
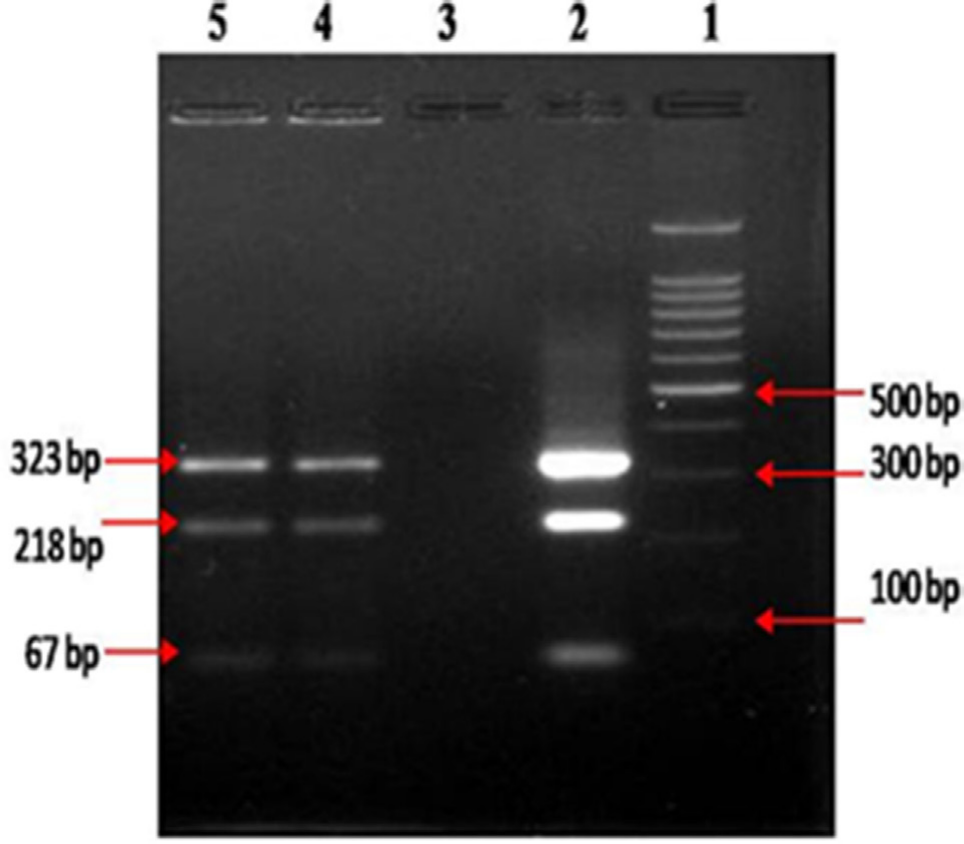
**Biotyping of “Indian Bison type” *MAP* bacilli, by *IS 1311* PCR_REA, from colonies developed at the end of 12 months of culture on HEY medium, columns 4 and 5**.

After 4 months of treatment, the patient exhibited improvement in his physical condition, reduction in stool frequency (two to three times a day from every half hour), and improvement in appetite. During the last months of treatment, the patient experienced continued improvement, with an increase in weight and further reduction in stool frequency (one to two times a day). Recovery from infection was complete following a year of treatment with no signs or symptoms of disease. The follow-up stool microscopy was negative for *MAP* (Figure 2B) and the follow-up post-treatment stool culture was negative for *MAP*. As mentioned, biotyping of colonies from cultures of stool showed the bacilli were the “Indian Bison type” of *MAP* (Figure 3). The ELISA was positive for *MAP* also, further confirmation that the patient had been infected with *MAP* (Table 2). The patient is now being monitored every 6 months for recurrence of infection and general health.

**Table 2:** **Screening of clinical samples (feces, blood, and serum) by multiple diagnostic tests for the presence of *MAP* infection at different days post-treatment**.

Tests	0th day	120 days	360 days
1. ELISA kit – serum^a^			
a. OD values	0.3255	0.7186	0.5826
b. Status of *MAP*	Negative	Positive	Positive
2. Microscopy – stool	+3 to +4	+2 to +1	Negative
3. Culture – stool^b^	Multi-bacillary	Pauci-billary	Negative
4. IS900 PCR-blood and stool^c^	Negative	Negative	Negative

*^a^Status of MAP infection as per S/P ratio (8) using “Indigenous ELISA kit”*.

*^b^MAP colonies took 1 year to grow*.

*^c^From DNA isolated directly from blood and stool samples*.

## Discussion

Recent studies have shown that humans like other species are susceptible to infection with *MAP*. The first evidence of susceptibility was obtained in studies demonstrating the presence of *MAP* in healthy subjects and patients with various diseases used as controls in the study of CD (2, 9–16). Most of these studies involved examination of small numbers of individuals, at a time when it was technically difficult to isolate *MAP* from tissues and fecal samples. Because it was thought that *MAP* is not pathogenic for humans, patients were not routinely tested for an infection with *MAP*. Our recent extensive survey of 42,400 individuals for the presence of *MAP*, across the social spectrum of populations living in India, a country where there is a high prevalence of *MAP* in domestic and wild animals, the food supply and environment, has documented that humans, regardless of health status and position in society, can be infected with *MAP* (1, 17–19).

Genomic studies documented that animals and humans were infected with a single biotype, the Bison biotype, the most prevalent biotype in India, with a signatory short single repeat (SSR), 7g4ggt, also found in the first isolates of *MAP* from humans in the United States of America (20). In addition to healthy subjects, the survey included patients with various illnesses, especially patients with intestinal disorders including CD (1, 2). Assays developed to test for infection in domestic animals were used to screen subjects for the presence of *MAP* (21). The microscopic assay of fecal samples was the most expedient way to identify subjects clearly shedding bacteria indistinguishable from *MAP*. In contrast to other studies, bacteria indistinguishable from *MAP* were detected in fecal samples of some patients with the clinical symptoms of CD. Follow-up assays with ELISA, culture, PCR with IS*900*, and biotyping of *MAP* colonies, using the IS*1311* PCR_REA and IS*1311* L2 PCR_REA method (5, 22), documented the isolates were the Indian Bison biotype of *MAP* (21, 23, 24). The survey also revealed that the bacterial load in stool was too low to be routinely detected by microscopy but could be detected by stool culture and specificity documented by IS900 PCR. A similar finding has been reported by a group of investigators in Italy (16).

The patient described in this report was from a small village in a rural region of Rajasthan state. Although it is not known how he became infected, *MAP* is endemic in livestock and the environment in this region. In this setting, exposure of the entire population could be from the contaminated environment, direct contact with livestock, or from the consumption of milk or milk products. Farmer families in India maintain a close contact with the domestic livestock, especially milking cattle, buffaloes, and goats, and often share living space with animals due to limited socioeconomic conditions. Studies on the bio-burden and biotyping of *MAP* infection in large populations of domestic livestock, wild ruminants, and other animals in the past 29 years have shown that *MAP* is widely prevalent and endemic in the domestic livestock population of the country (4, 19). In addition, as mentioned previously, mass screening of the human population in this part of India revealed a high level (>30.0%) of infection with *MAP* (1). Screening of milk and milk products also revealed a high bio-presence of *MAP* (18). Biotyping of *MAP* strains has consistently shown the presence of the dominant Indian Bison biotype as the most prevalent biotype of *MAP* detected in humans (4, 19).

As stated previously, one of the reasons why it has been so difficult to establish that *MAP* is the causative agent of CD in, at least, a subset of patients with CD has been that *MAP* has not been isolated from all patients. Also, the potential remains that more than one disease presents with the clinical features of CD. A recent study by Chiodini et al. has reported that, “CD may be differentiated into 2 distinct biotypes, based on the detection of bacterial genomic sequences and virulence genes within submucosal tissues” (25). This case provides additional evidence, supporting studies showing *MAP* is the causative agent in, at least, a subset of patients with CD (26) and that antibiotic therapy may lead to a cure of CD and clearance of infection with *MAP*. The results are consistent with findings from a group of *MAP* infected patients treated successfully by one of the coauthors, Dr. J. Todd Kuenstner, in the United States using a regimen of multi-antibiotic therapy combined with ultraviolet blood irradiation (UVBI) (27, 28). Of major importance, the results obtained in this report and the mass survey in India emphasize the need for recognition, at the international level, that *MAP* is a zoonotic pathogen and that it is a health risk for humans and livestock (29).

## Informed Consent

Written informed consent was obtained from the patient for treatment and for publication of this report and accompanying images.

## Author Contributions

SS, JK, WD, and JM designed and helped write the report; PA performed the treatment; KD and AT analyzed the ethical and public health study; DS and SJ collected the patient’s clinical data; and NK, JS, SG, and KC analyzed the data and wrote the paper.

## Conflict of Interest Statement

The authors declare there were no financial or commercial conflicts of interest associated with the report of the present study.
